# Real-world performance of susceptibility testing for cefiderocol: insights from a prospective multicentre study on Gram-negative bacteria

**DOI:** 10.1093/jacamr/dlae169

**Published:** 2024-10-24

**Authors:** Alvaro Irigoyen-von-Sierakowski, Azahara Ocaña, Rosa Sánchez-Mayoral, Emilia Cercenado, GEIRAS-SEIMC Study Group, Javier Fernández, Ana López, Cruz Villuendas, Antonina Arias, Juan Manuel García-Lechuz, Mª Isabel Cameo, Carlos González, Pilar Merino, Icíar Rodríguez-Avial, Esther Viedma, Gregoria Megías, Mª Pilar Ortega, Cristina Pitart, Cristina Colmenarejo, Marina Fernández, Marta Alonso, Ana Torralba, Felipe Pérez, Pablo Camacho, Carmen Guerrero, Caridad Sáinz de Baranda, Berta Fidalgo, Ángeles Sampere, Noelia Hernando, Fátima Galán, Sonia Paredes, Lisbeth Goncalves, María Rodríguez, Carlos Fuster, Xavier Mulet, Mª Carmen Fernández, Germán Bou, Julia Guzmán, Mª Dolores Quesada, Jun Hao Wang, David Navarro, Margarita Garau, Paz Díaz, Mª Carmen Gallegos, Mariela Martínez, Ángel Rodríguez, Isabel Cristina López, Mª Luz Asensio, Mª Eugenia Portillo, Amaya Oteiza, Amparo San Pedro, Genoveva Yagüe, Yolanda Gil, Laura Barrado, Mª Teresa Pérez, Patricia Pérez, Victoria Ortiz de la Tabla, Concepción Gimeno, Nuria Tormo, Antonia Sánchez, Paula Martí, Ana Martín, Laura Floren, Francisco Javier Chamizo, María García, Desirée Cordero, Pedro de la Iglesia, Salvador Giner, José Luis López-Hontangas, Isabel Sánchez-Romero, José Luis Barrios, Alba Rivera, Fe Tubau, Nieves Gutiérrez, Fernando García-Garrote, Jorge Calvo, María Siller, Cristina Seral, Amparo Coira, Eugenio Garduño, Francisco José Vasallo, Emma Padilla, Belén Viñado, Ana Collazos, María Simón, Mª Auxiliadora Semiglia, Teresa Alarcón, María Díez, Diego Domingo, Susana Ramón, Gemma Jiménez, Mª Victoria García, Lucía Puente, Álvaro Leal, Alvaro Irigoyen-von-Sierakowski, Azahara Ocaña, Rosa Sánchez-Mayoral, Emilia Cercenado

**Affiliations:** Department of Clinical Microbiology and Infectious Diseases, Gregorio Marañón General University Hospital, Dr. Esquerdo 46, Madrid 28007, Spain; Department of Clinical Microbiology and Infectious Diseases, Gregorio Marañón General University Hospital, Dr. Esquerdo 46, Madrid 28007, Spain; Department of Clinical Microbiology and Infectious Diseases, Gregorio Marañón General University Hospital, Dr. Esquerdo 46, Madrid 28007, Spain; Department of Clinical Microbiology and Infectious Diseases, Gregorio Marañón General University Hospital, Dr. Esquerdo 46, Madrid 28007, Spain

## Abstract

**Objectives:**

Cefiderocol is a novel siderophore-conjugated cephalosporin developed for the treatment of multidrug-resistant Gram-negative bacterial (GNB) infections. However, the current gold standard for cefiderocol susceptibility testing, broth microdilution (BMD) using iron-depleted cation-adjusted Mueller–Hinton broth, presents challenges for many microbiology laboratories. In this study, we evaluate the real-world performance of disc diffusion (DD) and a commercial BMD method (ComASP^®^) to test cefiderocol susceptibility in a series of isolates collected prospectively from severely ill patients in a multicentre study.

**Methods:**

The susceptibilities of 1472 isolates (632 Enterobacterales, 532 *Pseudomonas aeruginosa*, 84 *Acinetobacter* spp. and 224 *Stenotrophomonas maltophilia*) collected in 60 Spanish hospitals were analysed following the EUCAST 2023 and 2024 criteria. We assessed the performance of DD (cefiderocol 30 μg disc, Liofilchem) and a commercial BMD method (ComASP^®^ Cefiderocol, Liofilchem).

**Results:**

A total of 1408 and 1450 isolates were susceptible by DD and ComASP^®^ BMD, respectively. Overall, the agreement between both methods was 96.9%. Forty-four isolates were resistant by DD but susceptible by ComASP^®^ BMD, and two were susceptible by DD but resistant by ComASP^®^ BMD (*Acinetobacter baumannii* isolates). Adoption of the updated 2024 EUCAST DD breakpoints and areas of technical uncertainty (ATUs) led to a decrease in susceptibility among Enterobacterales (95.3% versus 92.6%).

**Conclusions:**

DD is a straightforward, rapid and accessible method for routine determination of cefiderocol susceptibility in real-world practice. ComASP^®^ BMD shows a high agreement with DD in susceptible isolates and may help to resolve DD interpretability concerns in isolates with susceptibility results within the ATU, but caution is warranted when testing resistant isolates.

## Introduction

Cefiderocol is a novel siderophore-conjugated cephalosporin developed for the treatment of infections caused by Gram-negative bacteria (GNB), including carbapenemase-producing Enterobacterales, *Pseudomonas aeruginosa*, *Acinetobacter* spp. and *Stenotrophomonas maltophilia*.

It was approved by the European Medicines Agency in 2020 for the treatment of infections caused by aerobic GNB with limited treatment options and by the FDA in 2019 for the treatment of complicated urinary tract infections, hospital-acquired bacterial pneumonia and ventilator-associated bacterial pneumonia.^1^

Cefiderocol has characteristics that give it the ability to bind to extracellular iron and this way to use active iron transporters to enter the bacterial cell. It can also passively enter bacterial cells through outer membrane porin channels.^1^ These characteristics also allow it to remain stable in the presence of most beta-lactamases and carbapenemases, including Class B metallo-beta-lactamases and OXA-23-like carbapenemases found in *Acinetobacter baumannii*.^2^

There are studies that show high rates of susceptibility to cefiderocol,^3,4^ but there is an increase in the number of reports showing cefiderocol resistance.^5,6^ A recent systematic review and meta-analysis reported low rates of cefiderocol resistance overall, varying from 0.4%, 1.4% to 3% in *S. maltophilia*, *P. aeruginosa* and Enterobacterales, respectively, but over 8% in *A. baumannii*.^7^

The current gold standard for cefiderocol susceptibility testing is MIC determination by broth microdilution (BMD) using cation-adjusted iron-depleted Mueller–Hinton broth (ID-CAMHB) due to non-reproducible MICs obtained by standard cation-adjusted Mueller–Hinton broth.^8,9^ This approach might be challenging for most clinical microbiology laboratories because it is time-consuming and difficult to implement routinely.

Moreover, as the EUCAST claimed in 2022, some commercially available assays have problems with accuracy, reproducibility, bias and/or, for some, skipped wells, making it difficult to interpret results in the area of technical uncertainty (ATU) by disc diffusion (DD). In fact, one of them (Sensititre^®^ microplates EUMDROXF; ThermoFisher Scientific, Cleveland, OH, USA) had to be withdrawn from the market for this reason (https://www.eucast.org/ast-of-bacteria/warnings).

More recently, two products using iron-depleted cation-adjusted Mueller–Hinton broth (ID-CAMHB) have been commercialized for determination of the MIC of cefiderocol: UMIC^®^ (Bruker Daltonics GmbH & Co. KG, Bremen, Germany) and ComASP^®^ (Liofilchem^®^, Roseto degli Abruzzi, Italy). However, the EUCAST has indicated that these panels also have problems with interpretability and reproducibility, and further work to elucidate the problems surrounding cefiderocol susceptibility testing is necessary.

There are results indicating that DD is a robust method for determining cefiderocol susceptibility;^1^ thus, the EUCAST recommends that laboratories use this first-line method, as it successfully predicts susceptibility and resistance outside the ATU. However, the current EUCAST cefiderocol breakpoint tables show zones of ATU that are difficult to interpret (21–23 mm for Enterobacterales and 20–21 mm for *Pseudomonas* spp.). In addition, there are no breakpoints for either *Acinetobacter* spp. or *S. maltophilia* (inhibition zone diameters ≥17 mm and ≥20 mm for the 30 µg cefiderocol disc correspond to MIC values below the PK–PD breakpoint of susceptibility (≤2 mg/L) for *Acinetobacter* spp. and *S. maltophilia*, respectively).^10^

The EUCAST has also published a reading guide for cefiderocol BMD.^11^

Most studies evaluating the activity of cefiderocol have focused on selected isolates with known resistance mechanisms, primarily NDM producers and multidrug-resistant *A. baumannii* isolates.^3,7,12,13^ However, there is a paucity of research examining the real-world performance of susceptibility testing for cefiderocol in routine microbiology laboratories using non-selected isolates.

As the majority of clinical microbiology laboratories only have the availability of DD and some commercialized BMD methods, it is important to assess the performance of these methods in real-world clinical practice.

Previous studies have shown high rates of major errors (MEs) and very major errors (VMEs) when comparing BMD with other techniques.^12,13^ Moreover, it remains unknown whether the application of the updated 2024 EUCAST cefiderocol breakpoints and ATU zones will lead to additional inconsistencies when performing comparative studies among different commercialized methods.

Consequently, there is still a need to overcome the uncertainty of susceptibility testing and ATU results in a way that is approachable for most clinical microbiology laboratories.

In this study, we evaluated real-world performance of DD and a commercial BMD method (ComASP^®^) to test cefiderocol susceptibility using the recently implemented EUCAST 2024 breakpoints in a series of non-selected, prospectively collected 1472 Gram-negative clinical isolates, including fermentative and non-fermentative isolates, from patients admitted to the ICUs and oncology wards of 60 hospitals in Spain. We also assessed the impact of the recent EUCAST 2024 changes in the cefiderocol DD breakpoints (in which the ATU intervals have been narrowed) relative to the 2023 EUCAST breakpoints.

## Materials and methods

### Bacterial isolates and quality control strains

We performed a multicentre study in which 60 Spanish hospitals participated. Starting in November 2021, at each participating centre, 30 consecutive GNB clinical isolates were collected from patients admitted to the ICU, haematology, oncology and paediatric wards during their stay in the hospital, including Enterobacterales (*Escherichia coli*, *Klebsiella pneumoniae*, *Enterobacter* spp., *Klebsiella* spp., *Citrobacter* spp., *Serratia* spp., *Proteus* spp., *Morganella morganii*, *Salmonella* spp., *Hafnia alvei* and *Raoultella ornithinolytica*), *P. aeruginosa*, *Acinetobacter* spp. and *S. maltophilia*. All the strains were received at our institution, where susceptibility testing was centralized. Only one isolate per infection episode and patient was accepted.

The genera with fewer than 50 isolates were included in the ‘Other Enterobacterales group’ [*Serratia* spp. (*N* = 49), *Klebsiella* spp. (*N* = 45), *Proteus* spp. (*N* = 33), *Citrobacter* spp. (*N* = 22), *M. morganii* (*N* = 10), *Salmonella* spp. (*N* = 4), *R. ornithinolytica* (*N* = 3) and *H. alvei* (*N* = 2)].

### Quality control strains

*E. coli* ATCC 25922 and *P. aeruginosa* ATCC 27853 were used as quality control strains with the cefiderocol target values defined by Matuschek *et al*.^1^

Quality control strains for both methods were included on each testing day.

### DD methodology

DD tests were performed with 30 μg of cefiderocol discs (Liofilchem^®^) on unsupplemented Mueller–Hinton agar (Biomérieux, France) using a 0.5 McFarland inoculum. Readings were performed after incubation at 35+/−1°C under aerobic conditions for 18+/−2 h. The results were interpreted according to the EUCAST breakpoints (v.14, 2024).^10^ To assess the impact of recent changes in the ATUs and cefiderocol breakpoints published in 2024 by the EUCAST, the results were also interpreted according to the 2023 EUCAST breakpoints (v.13, 2023). (www.eucast.org).

### MIC determination by BMD using the ComASP^®^ cefiderocol panel

For BMD testing, commercial ComASP^®^ Cefiderocol panels were used. Each panel contained 15 2-fold dilutions of dried antibiotics (0.008–128 µg/mL) and included ID-CAMHB. Starting from the same 0.5 McFarland inoculum used for the DD method, we followed the manufacturer’s instructions. Briefly, the 0.5 McFarland was diluted 1:20 in saline (Solution A). Then, 0.4 mL of Solution A was added to a tube of ID-CAMHB provided in the kit to obtain Solution B. Finally, we dispensed 100 mcl of Solution B into each well and covered the panel with the lid provided. Readings were performed after incubation at 35 ± 1°C under aerobic conditions for 18 ± 2 h following the BMD (EUCAST) reading guide (v.5.0, 2024).^11^ The results were interpreted only according to the 2024 EUCAST breakpoints (v.14, 2024),^10^ which are consistent with those from 2023 (v.13, 2023).

### Statistical analysis

Agreement rates and the number of isolates with discordant results between the two methods were evaluated. Agreement was defined as the percentage of DD test results that aligned with BMD ComASP^®^ within the same interpretive category (susceptible or resistant) according to the EUCAST criteria. Disagreement was defined as instances where one method classified an isolate as susceptible or resistant, while the other method placed it in the opposite category.

Stata/BE v17.0 (StataCorp. 2021. Stata Statistical Software: Release 17. College Station, TX, USA: StataCorp LLC) was used for the statistical analysis.

## Results

### Quality control strains

The ranges of inhibition zones and MICs for *E. coli* ATCC 25922 were 27–29 mm and 0.06–0.25 mg/L, respectively.

The ranges of inhibition zones and MICs for *P. aeruginosa* ATCC 27853 were 26–28 mm and 0.12–0.5 mg/L, respectively.

All these values were within the inhibition zone and MIC ranges established for these quality control strains (*E. coli* ATCC 25922: 24–30 mm and 0.06–0.5 mg/L; *P. aeruginosa* ATCC 27853: 23–29 mm and 0.06–0.5 mg/L).^1^

### Bacterial isolates

The 1472 BGN isolates included 632 (42.9%) Enterobacterales, 532 (36.1%) *P. aeruginosa*, 84 (5.7%) *Acinetobacter* spp. and 224 (15.2%) *S. maltophilia*. Overall, 1450 (98.5%) isolates were susceptible to cefiderocol according to BMD, while 1408 (95.7%) were susceptible to cefiderocol according to DD. The numbers of DD results included in the ATU zone for Enterobacterales and *P. aeruginosa* were 55 and 6, respectively. Sixteen each of *E. coli* and *K. pneumoniae* were the genera with the greatest number of isolates in ATU, followed by *Enterobacter* spp., with 14. The distribution of the isolates, number of ATU isolates and susceptibility to cefiderocol are described in Table 1.

**Table 1.** Distribution of the isolates and their susceptibility to cefiderocol determined by DD [2023 EUCAST breakpoints (v.13, 2023) and 2024 EUCAST breakpoints (v.14, 2024)] and ComASP^®^ BMD

Microorganisms	N	Susceptibility, n/N (%) [isolates in ATU *n*/*N*, %]		
		DD (EUCAST 2023 breakpoints)	DD (EUCAST 2024 breakpoints)	ComASP^®^ BMD
Total	1472	1425/1472 (96.8) [28/1472, 1.9]	1408/1472 (95.7) [61/1472, 4.1]	1450/1472 (98.5)
Enterobacterales	632	602/632 (95.3) [16/632, 2.5]	585/632 (92.6) [55/632, 8.7]	628/632 (97.8)
*E. coli*	261	256/261 (98.1) [4/261, 1.5]	253/261 (96.9) [16/261, 6.1]	260/261 (99.6)
*K. pneumoniae*	141	129/141 (91.5) [3/141, 2.2]	123/141 (87.2) [16/141, 11.4]	135/141 (95,7)
*Enterobacter* spp.	62	52/62 (83.9) [8/62, 12.9]	46/62 (74.2) [14/62, 22.6]	58/62 (93.4)
Other Enterobacterales	168	165/168 (98.2) [1/168, 0.6]	163/168 (97.0) [9/168, 5.4]	165/168 (98.2)
*P. aeruginosa*	532	520/532 (97.7) [12/532, 2.3]	520/532 (97.7) [6/532, 1.1]	531/532 (99.8)
*A. baumannii*	52	47/52 (90.4) [N/A]	47/52 (90.4) [N/A]	45/52 (86.5)
*Acinetobacter* spp. (other than *A. baumannii*)	32	32/32 (100) [N/A]	32/32 (100) [N/A]	32/32 (100)
*S. maltophilia*	224	224/224 (100) [N/A]	224/224 (100) [N/A]	224/224 (100)

The number of isolates in ATU is shown in brackets.

N/A, non-applicable; *N*, number of total isolates; *n*, number of detected isolates.

A good correlation between MICs and inhibition zone diameters was observed within the cefiderocol susceptible strains. Overall, among the isolates in the ATU zone, the agreement was 96.9%, with 46 isolates showing discordant results.

**Figure 1.**
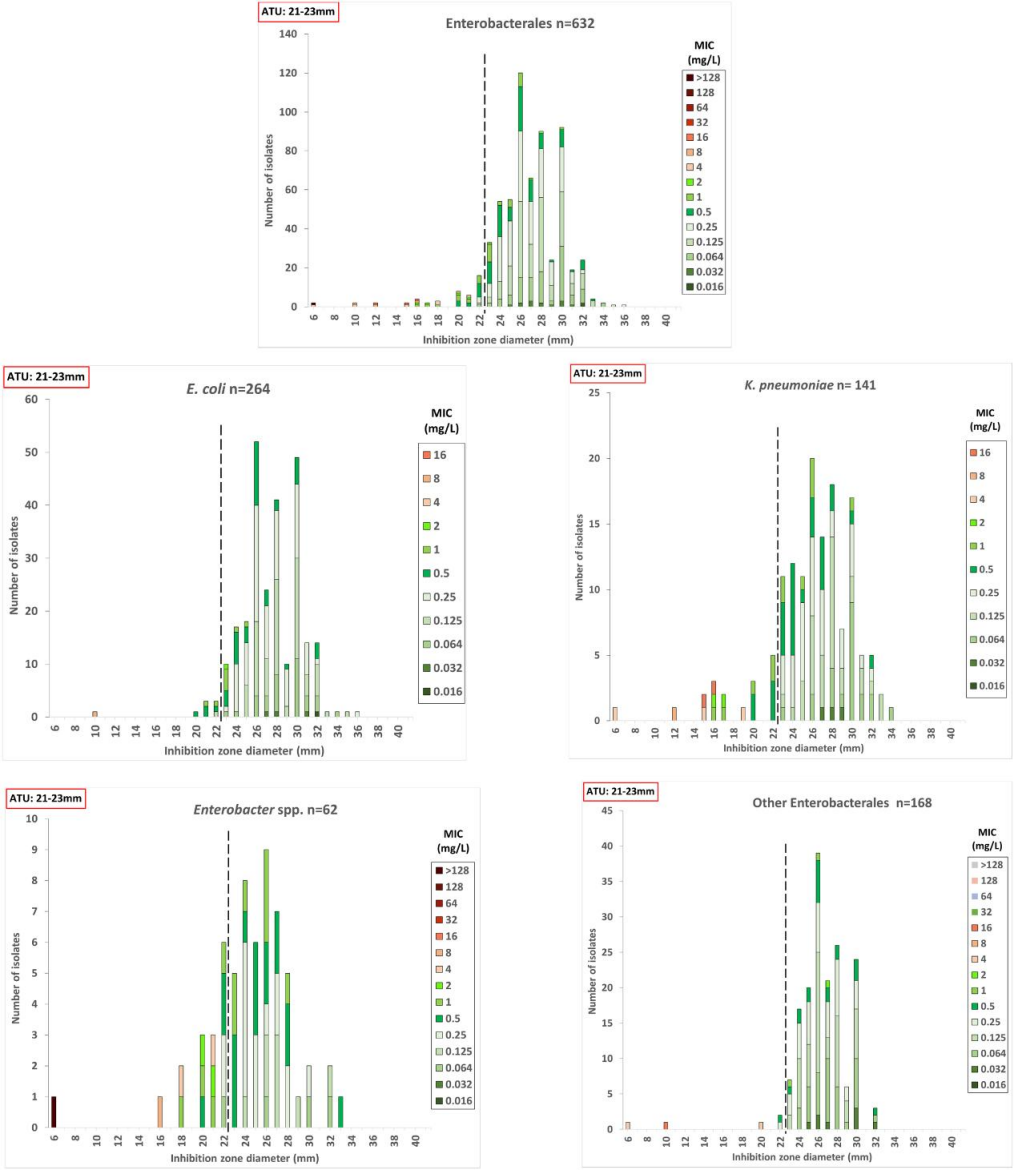
Distribution of inhibition zone diameter [30 μg discs (Liofilchem, Italy)] according to the MIC (mg/L) determined by ComASP^®^ cefiderocol panels (Liofilchem, Italy) for Enterobacterales. The coloured bars represent susceptible and resistant strains; the discontinuous vertical line marks the EUCAST zone diameter breakpoints (23 mm).

For Enterobacterales, the agreement was 94.8%, with 33 discrepancies. The agreement rates and the number of discordant results for the included species were 97.3% and 7 for *E. coli*, 91.5% and 12 for *K. pneumoniae*, 80.6% and 12 for *Enterobacter* spp. and 98.8% and 2 for other Enterobacterales, respectively. When excluding ATU isolates from the analysis, the agreement increased in all subgroups, while the number of mismatched results dropped (Table 2 and Figure 1).

**Table 2.** Agreement rates and number of discrepancies between the two methods including and excluding the isolates in the ATU, when comparing ComASP^®^ BMD with DD

	Agreement (susceptible or resistant) by ComASP^®^ BMD and DD (%)		Disagreement (*N*)			
			Susceptible by ComASP^®^ BMD and resistant by DD		Susceptible by DD and resistant by ComASP^®^ BMD	
	All included	ATU excluded	All included	ATU excluded	All included	ATU excluded
Overall	96.9	98.6	44	17	2	2
Enterobacterales	94.8	97.9	33	12	—	—
*E. coli*	97.3	99.6	7	1	—	—
*K. pneumoniae*	91.5	94.4	12	7	—	—
*Enterobacter* spp.	80.6	91.7	12	4	—	—
Other Enterobacterales	98.8	100	2	—	—	—
*P. aeruginosa*	97.9	99.0	11	5	—	—
*A. baumannii*	96.2	NA	—	NA	2	NA
Acinetobacter spp. (other than *A. baumannii*)	100	NA	—	NA	—	NA
*S. maltophilia*	100	NA	—	NA	—	NA

NA, non-applicable.

**Figure 2.**
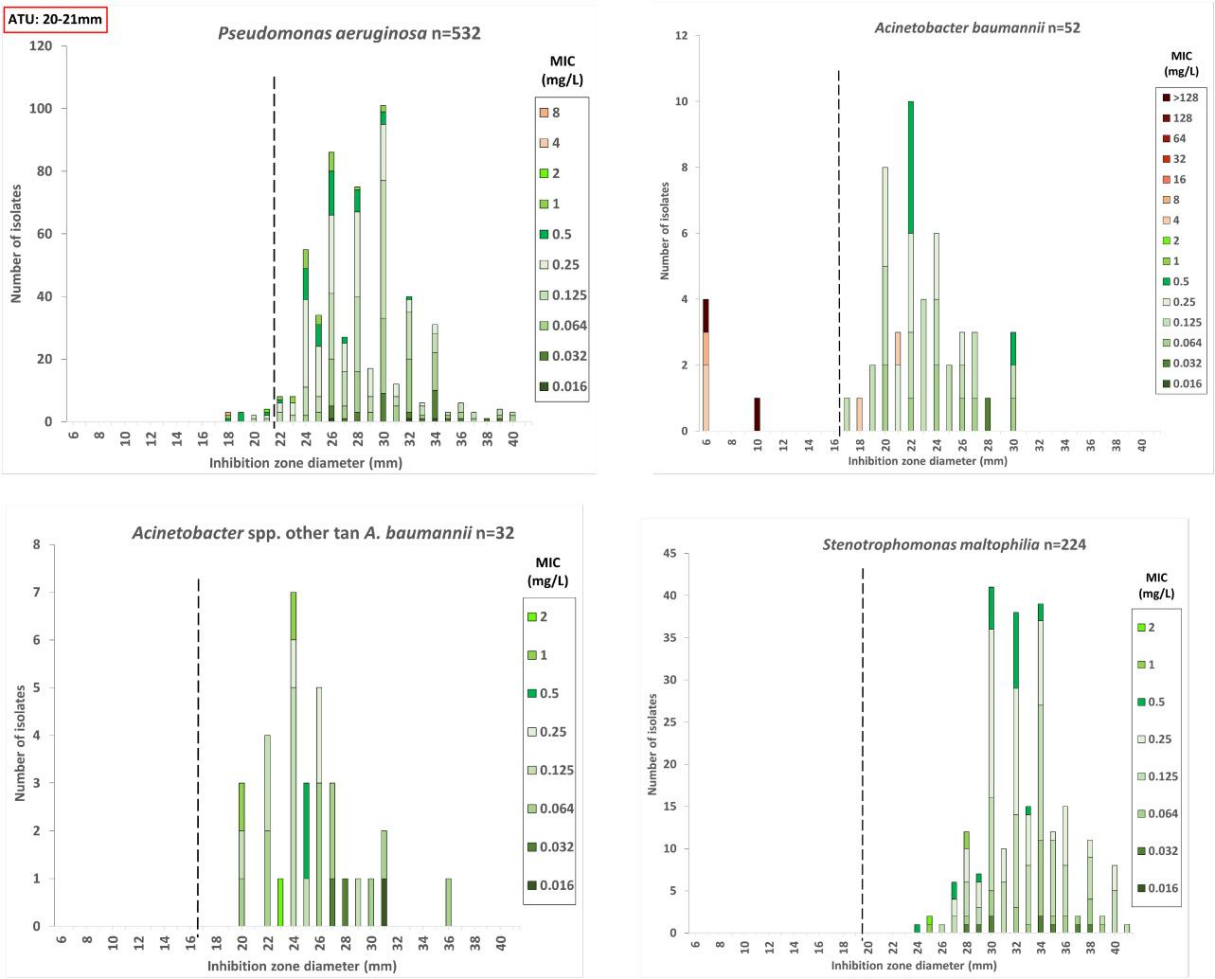
Distribution of inhibition zone diameter [30 μg discs (Liofilchem, Italy)] according to the MIC (mg/L) determined by ComASP^®^ cefiderocol panels (Liofilchem, Italy) for *P. aeruginosa*, *Acinetobacter* spp. and *S. maltophilia*. The coloured bars represent susceptible and resistant strains; the discontinuous vertical line marks the EUCAST zone diameter breakpoints (22 mm for *P. aeruginosa* and 17 and 20 mm PK–PD breakpoints for *Acinetobacter* spp. and *S. maltophilia*, respectively).

For *P. aeruginosa*, the agreement was 97.9%, which reached 99.0% once ATUs were excluded from the analysis, and 11 inconsistent results (Table 2 and Figure 2).

The agreement for *A. baumannii* was 96.2%. It was the only species where DD showed susceptibility, while the MIC method showed resistance in two isolates (Table 2 and Figure 2). Notably, both isolates had an MIC of 4 mg/L, which falls within the 2-fold dilution margin of error commonly accepted for BMD.

In the case of *S. maltophilia* and *Acinetobacter* spp. other than *A. baumannii*, all the isolates were susceptible by both methods, with an agreement of 100% and, consequently, no inconsistent results (Table 2 and Figure 2).

Table 1 shows variations in susceptibility based on the 2023 and 2024 EUCAST breakpoints. Adoption of the updated 2024 EUCAST DD breakpoints led to a decrease in susceptibility among Enterobacterales (95.3% versus 92.6%), notably affecting *Enterobacter* spp. (83.9% versus 74.2%) and *K. pneumoniae* (91.5% versus 87.2%). In addition, implementing the new ATU zones resulted in an increase in isolates falling within this zone for Enterobacterales (2.5% versus 8.7%), while a decrease was observed for *P. aeruginosa* (2.3% versus 1.1%).

## Discussion

The increase in bacterial resistance enhances the need for new effective antimicrobial alternatives. The wide spectrum of cefiderocol makes it an excellent option due to its ability to also act on carbapenemase-producing GNB and other multiresistant non-fermentative GNB. Nevertheless, the EUCAST standard method for cefiderocol susceptibility testing might not be easily performed in all clinical laboratories. In addition, difficulties in antimicrobial susceptibility reading and the range of ATU zones of DD described for Enterobacterales and *P. aeruginosa* make the categorization of the isolates difficult to interpret.^10^

The results of this study suggest that DD is an easy-to-perform and rapid method for routine determination of cefiderocol susceptibility in real world. The commercial ComASP^®^ BMD shows a high agreement with DD in susceptible isolates and may help to resolve DD interpretability concerns in isolates with susceptibility results within the ATU zones (see Table 2 and Figures 1 and 2).

As indicated above, our study showed an overall excellent agreement of DD with ComASP^®^ BMD, when testing cefiderocol susceptible isolates; these results are in accordance with those of other studies assessing the performance of DD for cefiderocol.^1,12–14^ In our study, the agreement rates were >90% for all genera except for *Enterobacter* spp., which had the lowest agreement rate (80.6%), which could be explained by the high number of ATU isolates in this group. Once the ATU isolates were removed from the analysis, the agreement rate for *Enterobacter* spp. reached 91.7%.

Regarding *A. baumannii*, two isolates were classified as by DD and resistant by ComASP^®^ BMD. This finding is consistent with the results of other studies that reported high rates of VMEs in this species when comparing DD with standard BMD.^12,13^

One of the strengths of this study lies in the inclusion of a high number of non-selected Gram-negative isolates from different Spanish hospitals that were recovered prospectively from severely ill patients. In addition, this study provides valuable insights into the impact of the newly approved 2024 EUCAST breakpoints and their implications. Our findings revealed that these changes resulted in an increase in resistant isolates when susceptibility testing was performed by DD, along with an increased number of isolates falling in the ATU zone and inconsistencies between DD and ComASP^®^ BMD. To the best of our knowledge, to date, studies comparing different cefiderocol AST methods have used the previous 2023 EUCAST breakpoints and have generally reported a strong agreement.^1–5,7–9,12,13,15–22^

Based on our findings, an isolate showing resistance to DD or falling within the zone of ATU should undergo testing using an alternative BMD method. A study comparing DD and BMD methods in *A. baumannii* suggested that DD results obtained using ID-CAMH-agar plates exhibit a significantly greater categorical agreement than DD results obtained using unsupplemented Mueller–Hinton agar plates.^12^ As the EUCAST DD breakpoints are established based on the use of unsupplemented Mueller–Hinton agar plates, some authors have suggested that inconsistencies between DD and BMD (performed in ID-CAMHB) may arise from the suboptimal performance of DD in non-ID Mueller–Hinton agar, as observed in our study.^12,19^

We advocate for the use of DD as a preliminary screening test in routine laboratory procedures due to its cost-effectiveness, simplicity and widespread availability. When isolates demonstrate clear susceptibility, the agreement between DD and BMD is generally excellent, obviating the need for further testing. However, when isolates show resistance or fall within the ATU zone, we recommend performing an alternative BMD method. In our experience, ComASP^®^ BMD has proven to be a reliable option for such patients.

One of the main limitations of our study is that only a few cefiderocol-resistant isolates were included, making the evidence for this subgroup insufficient. However, this approach reflects real-world clinical practice, where cefiderocol-resistant isolates are less frequently encountered in microbiology laboratories testing samples from severely ill patients. Besides, we did not perform the analysis based on the susceptibility of the isolates to other antibiotics, such as carbapenems. Bonnin *et al*.^16^ reported a categorical agreement rate of 77% when comparing DD with BMD in carbapenem-resistant Enterobacterales with high rates of VMEs even when excluding isolates from the ATU. Another study reported categorical agreement rates of 92% between the BMD method and DD for KPC-producing *K. pneumoniae*, with 16.7% MEs.^17^ Likewise, Devoos *et al*.^18^ assessed the performance of DD for multidrug-resistant *P. aeruginosa*, with categorical agreement rates ranging from 78% to 89%. This might indicate a potentially worse performance of the DD test in multidrug-resistant isolates, a problem that should be addressed in future studies.

Additionally, it is important to acknowledge that the isolates analysed in this study correspond only to those from Spanish hospitals, and as such, the epidemiological patterns observed may differ from those of other countries.

Another limitation of this study is that we did not perform AST by using the standard BMD method. This procedure is labour-intensive, time-consuming and challenging to implement in a clinical laboratory setting, and our aim was to compare two easy-to-perform techniques applicable in a real-world clinical microbiology laboratory routine. In addition, DD for cefiderocol AST has been validated by the EUCAST. Although the EUCAST has not yet validated ComASP^®^, several studies have compared this method with standard BMD measurements,^2,12,13,20^ including those of different bacterial species (Enterobacterales, *P. aeruginosa*, *Acinetobacter* spp. and *S. maltophilia*), and have demonstrated good performance on susceptible isolates but only acceptable in resistant isolates.^12,13,21–23^

To the best of our knowledge, this is one of the few and largest studies analysing both DD and a commercial BMD method to determine cefiderocol susceptibility and one of the first using the EUCAST 2024 breakpoints. Our study highlights the practical utility of DD as a straightforward, rapid and accessible method for routine determination of cefiderocol susceptibility, particularly valuable in settings where reference BMD is not available.

Additionally, the commercial susceptibility testing system (ComASP^®^ BMD) proves to be a feasible alternative that can be implemented in most clinical microbiology laboratories, particularly beneficial for resolving interpretability concerns in isolates falling within the ATU zones.
